# Feasibility and preliminary efficacy of a virtual reality intervention targeting distress and anxiety in primary brain tumor patients at the time of clinical evaluation: Study protocol for a phase 2 clinical trial

**DOI:** 10.1186/s12885-023-10671-2

**Published:** 2023-03-21

**Authors:** Amanda L. King, Alvina A. Acquaye-Mallory, Elizabeth Vera, Tito Mendoza, Jennifer Reyes, Macy L. Stockdill, Mark R. Gilbert, Terri S. Armstrong

**Affiliations:** Neuro-Oncology Branch, National Cancer Institute, National Institutes of Health, Bethesda, MD 20892 USA

**Keywords:** Virtual reality, Primary brain tumor, Distress, Anxiety

## Abstract

**Background:**

Primary brain tumor (PBT) patients experience higher levels of distress and anxiety than other solid tumor patients, particularly at the time of clinical evaluation when uncertainty about disease status is high (“scanxiety”). There is promising evidence supporting use of virtual reality (VR) to target psychological symptoms in other solid tumor patients, though PBT patients have not been studied extensively in this context. The primary aim of this phase 2 clinical trial is to establish the feasibility of a remote VR-based relaxation intervention for a PBT population, with secondary aims designed to determine preliminary efficacy of improving distress and anxiety symptoms.

**Methods:**

PBT patients (N = 120) with upcoming MRI scans and clinical appointments who meet eligibility will be recruited to participate in a single arm trial conducted remotely through the NIH. Following completion of baseline assessments, participants will complete a 5-min VR intervention via telehealth using a head-mounted immersive device while under supervision of the research team. Following the intervention, over the course of 1 month patients can use VR at their discretion with follow-up assessments done immediately post-VR intervention, as well as 1 week and 4 weeks later. Additionally, a qualitative phone interview will be conducted to assess patient satisfaction with the intervention.

**Discussion:**

Use of immersive VR is an innovative interventional approach to target distress and scanxiety symptoms in PBT patients who are at high risk for experiencing these symptoms leading into their clinical appointments. Findings from this study may inform design of a future multicenter randomized VR trial for PBT patients and may aid in development of similar interventions for other oncology populations.

**Trial Registration:**

Clinicaltrials.gov (NCT04301089), registered 9 March 2020.

**Supplementary Information:**

The online version contains supplementary material available at 10.1186/s12885-023-10671-2.

## Background

Psychological distress in cancer patients has been defined by the National Comprehensive Cancer Network (NCCN) as a multifactorial unpleasant emotional experience of a psychological, social, and/or spiritual nature that can interfere with the ability to effectively cope with the cancer diagnosis, its physical symptoms, treatment-related toxicities, and diagnostic imaging [1–3]. Distress, anxiety, and other psychological disorders may be more prevalent in PBT patients, compared to both the general population and those with non-CNS tumors [4, 5]. Once diagnosed, the overall prognosis for PBT patients remains poor, and they often have a difficult clinical course and a high symptom burden [6, 7]. Concomitant medications, particularly corticosteroids that are used to treat brain edema, may also contribute to worsening psychological symptoms for these patients [8]. The term “scanxiety” describes the distress related to often-debilitating anxiety solid tumor patients can experience in the period surrounding their diagnostic imaging studies and leading up to their clinic appointments [9]. Past research has shown that PBT patients experience significant uncertainty surrounding their illness and that alterations in mood (e.g. anxiety and depression) may be modifiers of the relationship between uncertainty and overall symptom burden [10]. Therefore, we propose that mitigating clinically significant distress and anxiety in PBT patients will result in improvements in their psychological and physical health.

The COVID-19 pandemic and associated infection mitigation procedures, including social distancing, lockdowns, travel bans, and changes to work practices, have imposed additional stress on cancer patients, as well as the general population [11, 12]. There is emerging evidence across the globe that fear of contracting COVID-19, the negative impact of social distancing and other mitigation procedures, and economic uncertainty are associated with higher levels of distress, anxiety, and depression within the general population [13–17]. Additionally, recent studies focusing on psychological symptoms in adult oncology patients during the pandemic [11, 18] have reported that over 30% of patients are experiencing high levels of stress, anxiety, and depression, as well as higher levels of loneliness and financial toxicity in those with severe psychological symptoms. While this data is primarily from breast and hematologic cancer populations, it is likely that there is a similar impact on psychological symptoms for PBT patients.

Virtual reality (VR) is a digital technology that offers immersive computer-graphic or video-based content of images and sounds that represent a real place or situation, which allows users to explore and interact with a virtual environment in a way that makes them feel actually present in that world [19–21]. VR has the potential to alleviate some of the negative aspects of illness by providing multisensory information and allowing individuals to “escape” to pleasant locations and more positive thoughts and emotions [22]. Past research has found VR technology to be efficacious in improving a variety of patient symptoms across both adult and pediatric populations [23–25], though relatively few studies have employed VR interventions to target symptoms in oncology populations, which is somewhat surprising. A systematic review of the literature focused on use of VR in adult and pediatric oncology populations [26] revealed some promising effects of VR on distress, anxiety, depression, and distraction, though small sample sizes and measurement heterogeneity across studies made applications of findings to PBT patients difficult. Further investigation is needed to determine the feasibility and efficacy of using VR technology to improve adverse psychological symptoms in PBT patients, which patients are most likely to benefit, and the underlying biological mechanisms by which this intervention might improve symptomatology. This study aims to address these important questions, which have to date been largely understudied within the brain tumor population.

### Study aims

The primary aim of this study is to evaluate the feasibility of using a VR relaxation intervention to target distress and anxiety symptoms in a PBT population at the time of clinical evaluation. Feasibility will be determined by the proportion of eligible patients who agree to participate in the study, the proportion who complete the VR intervention, the proportion of data completeness, incidence of grade 3 or higher device-related adverse effects, and patient satisfaction with the intervention.

Secondary aims of this study including the following:

1. To assess the effects of the VR intervention on self-reported acute (immediate post-intervention) and subacute (1 week to 4 weeks post-intervention) distress and anxiety symptoms.
2. To determine if the effects VR has on distress and anxiety are more pronounced in those with high distress (based on NCCN Distress Thermometer [DT] cut-off score of ≥ 5) compared to those with low distress (based on DT cut-off score of < 5).
3. To determine if the effects VR has on distress and anxiety are more pronounced in those individuals not on systemic corticosteroids (CS) compared to those who are on active CS therapy.

There are additional exploratory aims of this trial that include the following:

1. To explore the correlations between biological stress measures (as measured by salivary cortisol, dehydroepiandrosterone-sulfate [DHEA-S], and alpha amylase [sAA]) with patient-reported outcomes (PROs)
2. To explore the effects of a VR intervention on PROs collected on the Neuro-Oncology Branch (NOB) Natural History Study (NHS), including mood disturbance, symptom burden and interference, health-related quality of life (HRQoL), and cognitive function
3. To explore the impact of loneliness and financial toxicity on distress and anxiety symptoms during the COVID-19 pandemic
4. To determine the proportion of patients with adjustment disorder (AjD) in a PBT population to assess the potential utility of VR for improving AjD-related symptoms

## Methods

### Study design

**Fig. 1:**
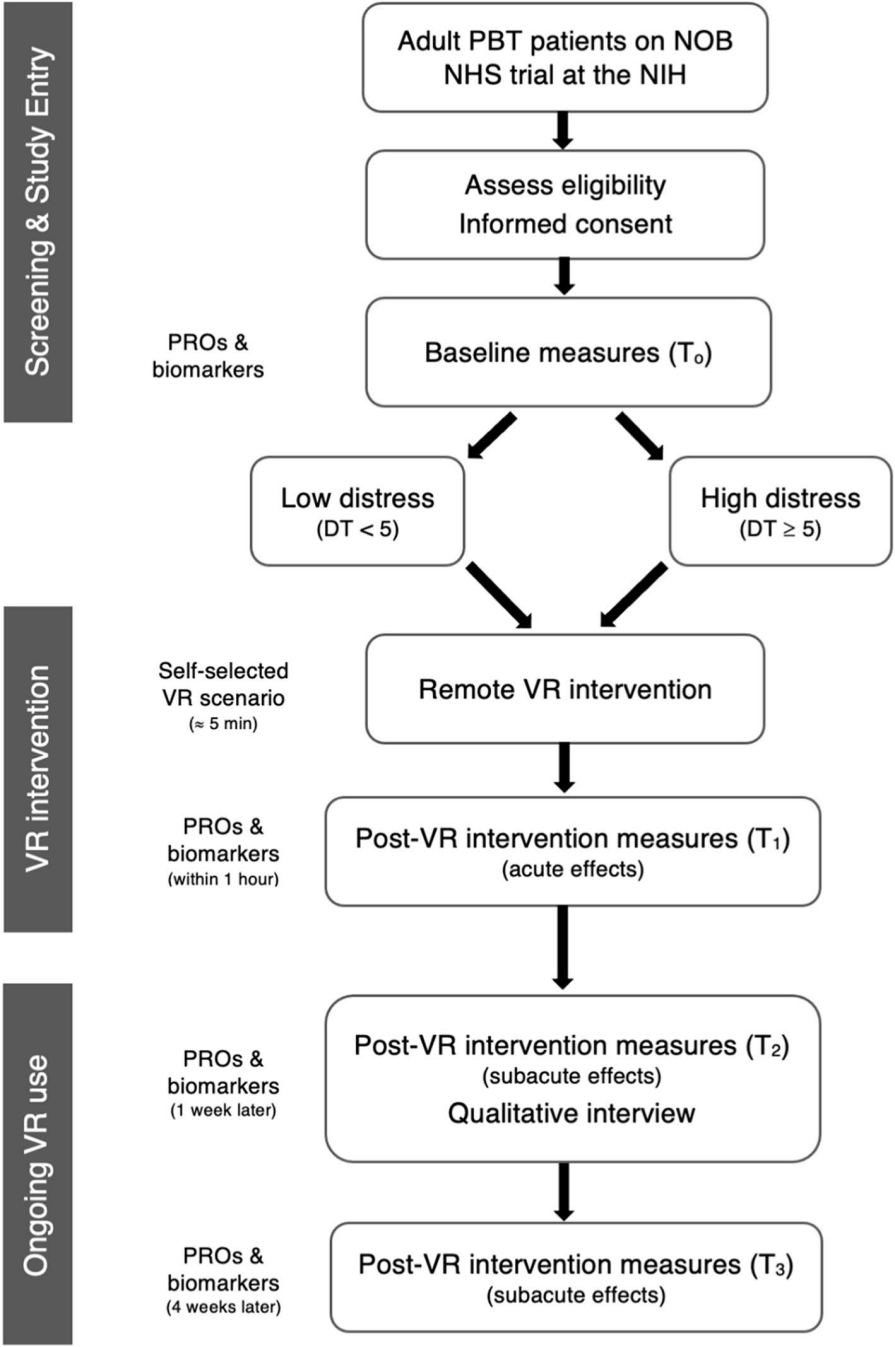
Phase 2 study protocol flow diagram. Adult PBT patients will be recruited from the NOB NHS trial at the NIH. Patients who meet eligibility criteria will be recruited via email or in clinic and will complete informed consent to join the study. Baseline PROs and salivary stress biomarkers will be collected and patients will be stratified into low vs. high distress groups (based on DT scores). Research staff will meet with patients via telehealth to complete the initial VR intervention where participants will self-select a scenario to complete, followed by post-VR intervention assessments to assess acute effects. Patients can continue VR use at home for the remaining month on study and repeat post-VR intervention assessments will be collected at the 1 week and 4 weeks timepoints to assess subacute effects. A qualitative phone interview will be conducted 1 week following the initial VR intervention to assess patient satisfaction

This is a phase 2 clinical trial with a single arm experimental design, shown in Fig. 1, which will evaluate the feasibility of using a VR relaxation intervention to target distress and anxiety symptoms for PBT patients at the time of clinical evaluation. Due to the COVID-19 pandemic, all aspects of this study will be conducted remotely through telehealth meetings with participants and use of VR in their home environment. Following collection of baseline assessments, participants will complete a remote VR intervention with members of the research team prior to their MRI scan and clinic or telehealth appointments. Post-intervention assessments will be collected immediately after the initial VR intervention, as well as 1 week and 4 weeks later, in order to explore acute and subacute effects of the intervention on distress and anxiety symptoms. Device-related adverse effects will be assessed throughout the participation period and a qualitative interview will capture patient satisfaction with the intervention. The schedule of enrollment, intervention, and assessments for this study is shown in Table 1.

**Table 1:**
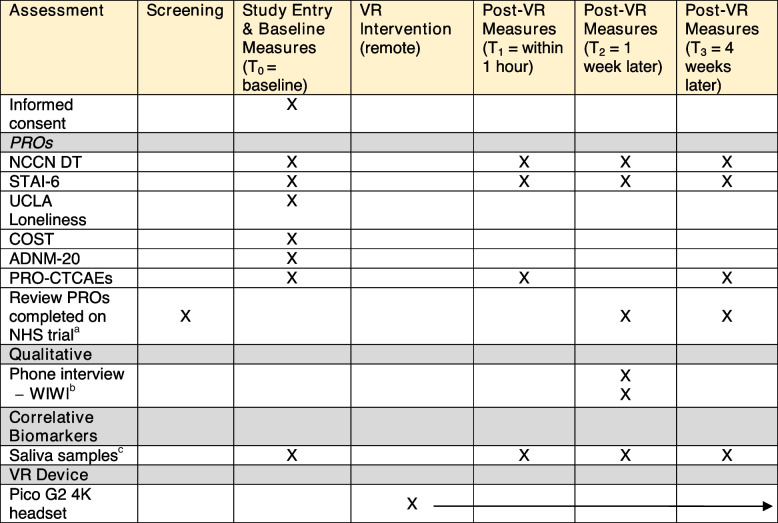
Schedule of enrollment, intervention & assessments for VR study

*Abbreviations: VR* Virtual reality, *PROs* Patient-reported outcomes, *NCCN DT* National Comprehensive Cancer Network Distress Thermometer, *STAI-6* State-Trait Anxiety Inventory (6-item), *PRO-CTCAEs* Patient-Reported Outcomes version of the Common Terminology Criteria for Adverse Events, *UCLA* University of California Los Angeles, *COST* Comprehensive Score for Financial Toxicity, *ADNM-20* Adjustment Disorder-New Module (version 20), *WIWI* Was It Worth It, *DHEA-S* Dehydroepiandrosterone-sulfate, *sAA* Salivary alpha amylase

^a^MDASI-BT, PROMIS®-Anxiety and PROMIS®-Depression Short-Forms, Neuro-QoL™ and EQ-5D questionnaires will be completed as part of the NHS trial

^b^WIWI questionnaire will be administered verbally during the qualitative phone interview

^c^Salivary hormone collection kits will be mailed to participants. This is an optional collection at the discretion of the patient and investigator

### Setting

This feasibility trial is being conducted at the NOB at the National Cancer Institute (NCI) within the National Institutes of Health (NIH) in Bethesda, MD, United States. The protocol for this study was reviewed and approved by the NIH Institutional Review Board and all methods will be carried out in accordance with relevant guidelines and regulations for human subjects protections. This trial was initially launched in March 2020, though due to the COVID-19 pandemic recruitment for this study did not begin until March 2021. Study recruitment is ongoing and is expected to be completed in 2023.

### Participants and recruitment

The study population will be comprised of patients who are actively enrolled on the NOB NHS trial (NCT02851706), which prospectively collects biological, clinical, and PROs data for individuals with primary CNS tumors [27]. Participants will be screened for eligibility based on pre-defined criteria, which are outlined in Table 2. The investigators will screen patients for eligibility through review of their clinic notes in the NIH electronic medical record (EMR) and through discussion with the clinical teams. Recruitment methods will include approaching patients during clinic or telehealth visits, or via email reach-outs using a study flyer (see Supplemental Fig. 1) that highlights key aspects of the trial. Interested patients who meet eligibility criteria will be consented remotely via telehealth.

**Table 2:** Inclusion and exclusion criteria for VR study

**Inclusion Criteria **	**Exclusion Criteria **
1. Diagnosed with PBT tumor (brain + spine disease permissible)	1. Lack of definitive tissue diagnosis (no past surgery or biopsy)
2. Enrolled on NOB NHS trial	2. Recent cranial surgery ≤ 2 weeks prior to VR intervention
3. Age ≥ 18 years old	3. Scalp wound healing issues that might interfere with VR headset use
4. Able to speak & understand English	4. Pre-existing diagnosis of epilepsy (prior to brain tumor diagnosis)
5. Able to understand & sign informed consent	5. Diagnosis of GAD, PTSD, claustrophobia, or panic disorder
6. Can reliably self-report symptoms (based on clinician assessment)	6. Hypersensitivity to motion or currently experiencing severe nausea
7. Upcoming clinic or telehealth appointment with MRI scan	7. Visual deficits, including hemianopsia, diplopia, and agnosia, that may interfere with VR experience
8. Reported distress ≥ 1 on past symptom questionnaires	

^a^*Abbreviations*: *PBT* Primary brain tumor, *NOB* Neuro-Oncology Branch, *NHS* Natural History Study, *MRI* Magnetic resonance imaging, *VR* Virtual reality, *GAD* Generalized anxiety disorder, *PTSD* Post-traumatic stress disorder

### Sample size considerations

The primary aim of this study is to determine the feasibility of implementing a VR intervention that aims to reduce distress and anxiety symptoms in PBT patients. This study will be considered successful if the following feasibility metrics are met: 80% of approached eligible patients agree to participate, 70% compliance with VR headset use during the initial intervention, 70% of required data points are completed, no grade 3 or higher device-related adverse effects (AEs), and high patient satisfaction with the intervention determined by the qualitative interview and Was It Worth It (WIWI) questions.

While feasibility can be assessed with small samples, the sample planned for this study was calculated to also be able to address the secondary and exploratory aims as well as the primary feasibility aim. Assuming that approximately 80% of patients approached for this study are eligible, a two-sided 95% confidence interval around the expected proportion of eligible patients will have a width of ± 7.8%. To account for 10% attrition of participants during the trial, we will plan to recruit a total of 120 patients for this study. In order to ensure adequate representation of high vs. low distress individuals and those on vs. off CS therapy to address the secondary aims of this study, we will aim to enroll approximately 20% of the sample with high distress and active CS therapy (*N* = 24) and approximately 80% with low distress and no CS use (*N* = 96).

### VR Intervention

Research staff will orient study participants to the VR headset in a telehealth meeting demonstrating fit, use, and navigation of the virtual environments. Once all baseline assessments have been completed and participants feel comfortable with use of the VR headset, they will complete the VR intervention under remote supervision by research staff. For the purposes of standardizing duration of the VR intervention across individuals, participants will self-select a scenario that is approximately 5 min in duration and they will not be permitted to change their scenario once they have begun. Research staff will remain in the telehealth meeting with the participants during the VR intervention to monitor for any issues or device-related AEs that may occur.

#### VR device

The Pico G2 4 K is a lightweight, stand-alone VR headset that comes with an orientation-tracked controller and does not require a smartphone or a PC to operate. This headset can be operated via “gaze mode” or “controller mode” where the user can make selections on the virtual platform by either directing their gaze at a particular item or with use of the handheld remote controller. Additionally, there is a breath shield attachment on the front of the device that has the ability to detect breathing patterns of the user and will change the virtual environment experienced if a breath-based scenario is chosen.

The VR software loaded on headset, termed SootheVR™, was designed by AppliedVR™ for therapeutic use within clinical populations and aims to improve adverse symptomatology and promote relaxation. There are a total of 41 scenarios on the VR headset that fall within 3 main categories: 1) Dynamic Breathing, 2) Guided Relaxation, and 3) Instant Escape. The Dynamic Breathing scenarios utilize the breath shield attachment and guide the patient to take slow, deep breaths as the environment changes based on their breathing pattern. Guided Relaxation scenarios provide meditative, calming environments that allow patients to practice mindfulness coping strategies. Instant Escape scenarios provide distraction from unpleasant symptoms where patients can observe ocean environments, travel to beautiful places, or play interactive games. While there are several interactive games on the VR device, these are not options for participants to choose during the VR intervention since they tend to be more stimulating than relaxing.

#### Post-intervention VR use

Following the remote VR intervention, participants will be permitted to use the VR headset as often as they desire and can choose any scenario available while using the device at home (including games). Research staff will conduct weekly check-ins with participants, either via phone or email, in order to determine how often participants are using VR, what types of scenarios they have been doing and which have been most helpful, to address any technological issues, and to ask about device-related AEs. Other members of the household are permitted to use the VR headset and we will encourage the participants to inform us if this occurs, though we will not collect data from those individuals.

### Study outcome measures

#### Patient-reported outcomes

##### NCCN DT

The DT is a 1-item, 11-point Likert scale that is represented on a visual graphic of a thermometer that ranges from 0 (no distress) to 10 (extreme distress), with patients reporting their level of distress over the preceding week (including the day of assessment). Individuals who report high levels of distress can be given the accompanying problem list that identifies commonly reported problems related to the cancer experience. The DT has been validated in numerous oncology populations and has demonstrated validity and reliability against other validated legacy instruments [28, 29]. In past DT research within oncology, cut-off scores indicating clinically significant distress have varied, but the majority support a DT cut-off score of either 4 or 5 [30, 31], which we will align with.

##### STAI-6

The STAI-6 is one of the most commonly used measurement tools for anxiety and has been well-validated in past research [32, 33]. Despite its popularity and sound psychometrics in past research, the legacy instrument is rather lengthy, consisting of 40 items total with 2 subscales: state anxiety, known as the S-scale (how anxious one feels in that moment) and trait anxiety, known as the T-scale (how anxious one generally feels). For the purposes of this study, we are more interested in state anxiety and how a VR intervention might be effective at reducing this kind of transient anxiety. We will use the STAI-6 S-scale developed by Marteau and Bekker [32], given it was validated in outpatient participants and tends to perform best from a psychometric standpoint compared to other short versions of the instrument.

##### UCLA Loneliness Scale

The UCLA Loneliness Scale is a widely used instrument in the literature with well-established psychometrics in a variety of clinical populations [34], including oncology [35]. It utilizes a 20-item scale that is designed to measure one’s subjective feelings of loneliness (10 items) as well as feelings of social isolation (10 items), with participants rating each item on a Likert scale from 1 (Never) to 4 (Often). While short-forms of this instrument have been developed, they do not perform as well from a reliability standpoint, therefore we will be utilizing the full 20-item version of the UCLA Loneliness Scale for this study.

##### COST

The COST instrument will be used to measure financial toxicity, which has been defined as “the distress patients can experience related to the high costs of cancer treatment and subsequent economic challenges (such as loss of income)” [36]. This is an 11-item instrument using a 5-point Likert scale ranging from 0 (Not at All) to 4 (Very Much) asking patients about the financial impact that their disease and treatment has had on their lives, with higher scores indicated higher distress related to financial concerns.

##### ADNM-20

The Adjustment Disorder New Module 20-item instrument (ADNM-20) will be used to screen for adjustment disorder (AjD) in this study population. This instrument measures AjD as a stress response disorder and consists of 2 parts: a stressor list and an item list [37]. The stressor list captures a broad range of acute (e.g. divorce, moving) and chronic (e.g. serious illness, family or work conflicts) stressors that have occurred over the last two years. The item list measures the symptoms in response to the most distressing event identified by the participant. Individuals indicate on a 4-point Likert scale, ranging from 1 (never) to 4 (often), how often they have experienced different symptoms of an adjustment disorder in the past 2 weeks.

##### PRO-CTCAEs

PRO-CTCAE is a patient-reported outcome measurement system that was developed by the NIH in order to capture the symptomatic adverse events in patients on oncology clinical trials [38]. This instrument allows us to select, but not be limited to, the symptoms that are anticipated with use of the VR headset based on past clinical research and experience with the technology, including nausea, vomiting, dizziness, and headache, with the option to report other unanticipated symptoms.

#### Qualitative assessment

A brief, 7-item semi-structured questionnaire (see Supplemental Fig. 2A) developed by a qualitative researcher will be used during a phone interview with participants 1 week following the remote VR intervention. The purpose of the interview is to allow the participants to share their experiences with VR and to provide feedback about the experience, as well as about the headset itself. Additionally, information regarding any side effects or adverse symptoms from using the VR headset will be discussed. There is also a question related to their experience with having a brain tumor during the COVID-19 pandemic and any psychological symptoms they are having during this time. The interview will conclude with 4 Was It Worth It (WIWI) questions (see Supplemental Fig. 2B) being asked verbally in order to assess satisfaction with the intervention. The phone interview will be recorded, and the content transcribed in preparation for qualitative thematic analysis.

#### Correlative biomarkers

There will be optional collection of salivary cortisol, DHEA, and sAA as correlative biomarkers, which together represent activity of the neuroendocrine stress regulatory systems. By measuring these stress biomarkers and correlating their activity with self-report psychological measures, we hope to gain a more holistic picture about the efficacy of stress reducing interventions for PBT patients and what biological mechanisms are involved. Supplies to collect saliva will be mailed to the patient’s home along with the VR headset prior to the VR intervention and detailed saliva collection instructions will be provided.

### Data management and monitoring

The PROs data from the questionnaires will be collected via the Scribe electronic interface using links emailed to participants at the 4 study timepoints (baseline, immediate post-VR intervention, 1 week post-VR intervention, and 4 weeks post-VR intervention). This data, along with participant demographic and clinical data, will be exported into a password-protected internal database and audited for errors by trained data analysts. To protect participant confidentiality, patient identifiers will be stored in a separate location from the research data and only the key study personnel will have access to identifiers.

Adverse events related to use of the VR device will be captured via the PRO-CTCAEs questionnaires and through patient report during weekly check-ins with the research team. In the event that the participant complains of any adverse effects, either during the intervention or with ongoing VR use at home, they will be instructed to remove the VR headset and allow time to recover from the symptoms. The research team will be notified and if symptoms persist, despite a break from using the VR device, they will be removed from therapy and will complete follow-up PROs assessments, per investigator and clinician discretion.

### Statistical analysis

#### Primary aim

To evaluate the feasibility of the VR intervention, we will use descriptive statistics to summarize rates of recruitment and retention, data completion, compliance, device-related adverse events, and participant satisfaction. We will calculate total scores, subscale scores, and/or *t*-scores (when applicable) for the PRO measures, completed at varying timepoints. The quantity of missing data, variability of data over time, and trends over time will be especially important to report, which will be summarized by time point quantitatively and graphically. Patients who drop out will also be compared to those who are retained, based on their demographic characteristics and other baseline assessments using *t*-tests or Wilcoxon rank sum tests. A multiple logistic regression model will be constructed to identify variables associated with dropping out of the study, which will help determine the characteristics of patients who are recruited to the study and those who are retained throughout the participation period. This will help guide future recruitment efforts and may identify types of patients who may require special attention to minimize attrition.

We will use descriptive statistics to report how patients rate their responses on various PRO measures. For the NCCN DT, we will report the proportion of patients who scored ≥ 5 indicating moderate-severe distress. We will report subscale scores for the MDASI-BT and the proportion of patients moderate-severe ratings on the 0 to 10 numeric rating scale of each symptom item. *T*-scores from the PROMIS® Anxiety & Depression Short Forms and Neuro-QoL™ instrument will also be reported. Additionally, we will report participant satisfaction with the VR intervention using descriptive statistics from the WIWI questionnaire, as well as through qualitative thematic analysis from participant responses during the semi-structured phone interview.

#### Secondary aims

To determine the effects of a VR relaxation intervention on self-reported acute and subacute distress, we will fit a linear mixed model with patients as random effect and time as fixed effect using scores from the DT as the dependent variable. To quantify the acute VR effects, we will calculate effect size difference between baseline scores and immediate post-VR intervention scores. To quantify the subacute VR effects with continued use at home, we will calculate effect size differences in distress scores between baseline and weeks 1 and 4 post-VR intervention. A similar approach will be taken to analyze effects of the VR intervention on acute and subacute anxiety, using the STAI-6 as the dependent variable in a linear mixed model. Similar effect size calculations will be made comparing anxiety scores between the baseline, immediate post-VR intervention, and weeks 1 and 4 post-VR intervention timepoints.

To determine if the effect VR has on distress and anxiety is more pronounced in high vs. low distress individuals, we will calculate difference scores in distress between the baseline and immediate post-VR intervention timepoints. We will then perform an independent *t*-test on the distress difference scores using 2 groups. We will repeat the analyses using the same groups, but with changes in anxiety as the dependent variable. To determine if the effect VR has on distress and anxiety is more pronounced in those who are not on systemic CS therapy vs. those on active therapy, we will calculate difference scores in distress and anxiety based on the baseline and immediate post-VR intervention timepoints. We will then perform an independent *t*-test on the distress difference scores using 2 groups, based on whether or not they are on CS therapy.

## Discussion

This phase 2 clinical trial aims to evaluate the feasibility and preliminary efficacy of an immersive VR-based relaxation intervention to target distress and anxiety symptoms in PBT patients at the time of neuroimaging and clinical appointments. Within this population, it has been reported that psychological needs of patients are highly unmet [5] and there are few non-pharmacological interventions available to target these symptoms [26]. VR interventions for adverse symptomatology have shown efficacy in other solid tumor populations, but to our knowledge there are no known studies targeting the critical time leading into MRI scan and clinical appointments, which is a very distressing time for PBT patients. Our working hypotheses are as follows: 1) use of VR could elicit a combination of distraction and promotion of the relaxation response, both of which may blunt physiological stress pathways, and 2) cardiac coherence breathing and distraction in immersive VR environments could decrease stress and improve psychological symptoms. The results of this study will provide evidence of the feasibility of this interventional approach and preliminary evidence to support or refute our hypotheses.

A main limitation for this trial is its single-center, non-randomized design. There is clearly a need for multicenter, large-scale randomized VR trials to establish the benefits of VR-based relaxation scenarios on psychological symptoms, but before launching these efforts it is important to demonstrate feasibility and preliminary efficacy in this population. Another limitation is related to conducting this study during a global pandemic, which may have an impact on study feasibility. To counter this, we have varied recruitment approaches in place and weekly check-ins with enrolled patients to encourage data completion and safe use of the VR headset, which we anticipate will positively impact the feasibility of this study.

In conclusion, use of immersive VR is an innovative interventional approach to target distress and anxiety symptoms in PBT patients who are at high risk for experiencing these symptoms leading into their appointments. By sharing our feasibility trial protocol with the research community, we hope to aid in the development of similar interventions that target psychological symptoms for oncology patients, particularly those that can be done remotely. The results of this study will inform the design of a larger multicenter randomized controlled trial in the future to improve mood and quality of life for this population.

## Supplementary Information

**Additional file 1: Supplemental Figure 1.** Recruitment flyer for VR study. **Supplementary Figure 2.** Qualitative assessment of patient satisfaction with VR intervention.

## Data Availability

The study protocol for this research is posted on ClinicalTrials.gov and additional information about the trial is available from the corresponding author on reasonable request.

## Abbreviations

PBT: Primary brain tumor
VR: Virtual reality
MRI: Magnetic resonance imaging
NIH: National Institutes of Health
NCCN: National Comprehensive Cancer Network
CNS: Central nervous system
DT: Distress Thermometer
CS: Corticosteroids
DHEA-S: Dehydroepiandrosterone sulfate
sAA: Salivary alpha amylase
PROs: Patient-reported outcomes
NOB: Neuro-Oncology Branch
NHS: Natural History Study
HRQoL: Health-related quality of life
AjD: Adjustment disorder
EMR: Electronic medical record
AEs: Adverse events
WIWI: Was It Worth It questions
PRO-CTCAEs: Patient-Reported Outcomes version of the Common Terminology Criteria for Adverse Events
MDASI-BT: MD Anderson Symptom Inventory – Brain Tumor
STAI-6: State-Trait Anxiety Inventory – 6 item
