# Supplementary material for: Feasibility and preliminary efficacy of a virtual reality intervention targeting distress and anxiety in primary brain tumor patients at the time of clinical evaluation: Study protocol for a phase 2 clinical trial
Source: BMC Cancer. 2023 Mar 21;23:262. doi: 10.1186/s12885-023-10671-2 (PMC10030076; doi:10.1186/s12885-023-10671-2)
Supplement: Supplementary file 1 — Additional file 1: Supplemental Figure 1. Recruitment flyer for VR study. Supplementary Figure 2. Qualitative assessment of patient satisfaction with VR intervention. [file 12885_2023_10671_MOESM1_ESM.docx]

**Title:** Feasibility and preliminary efficacy of a virtual reality intervention targeting distress and anxiety in primary brain tumor patients at the time of clinical evaluation: Study protocol for a phase 2 clinical trial

**Authors:** Amanda L. King^1^, Alvina A. Acquaye^1^, Elizabeth Vera^1^, Tito Mendoza^1^, Jennifer Reyes^1^, Macy Stockdill^1^, Mark R. Gilbert^1^, Terri S. Armstrong^1^

**Affiliations:** ^1^Neuro-Oncology Branch, National Cancer Institute, National Institutes of Health

**Correspondence:** Amanda L. King, PhD, APNP-BC

Neuro-Oncology Branch, National Cancer Institute, National Institutes of Health

9030 Old Georgetown Road, Room B03A

Bethesda, MD, 20892

*Email:* [amanda.king2@nih.gov](mailto:amanda.king2@nih.gov)


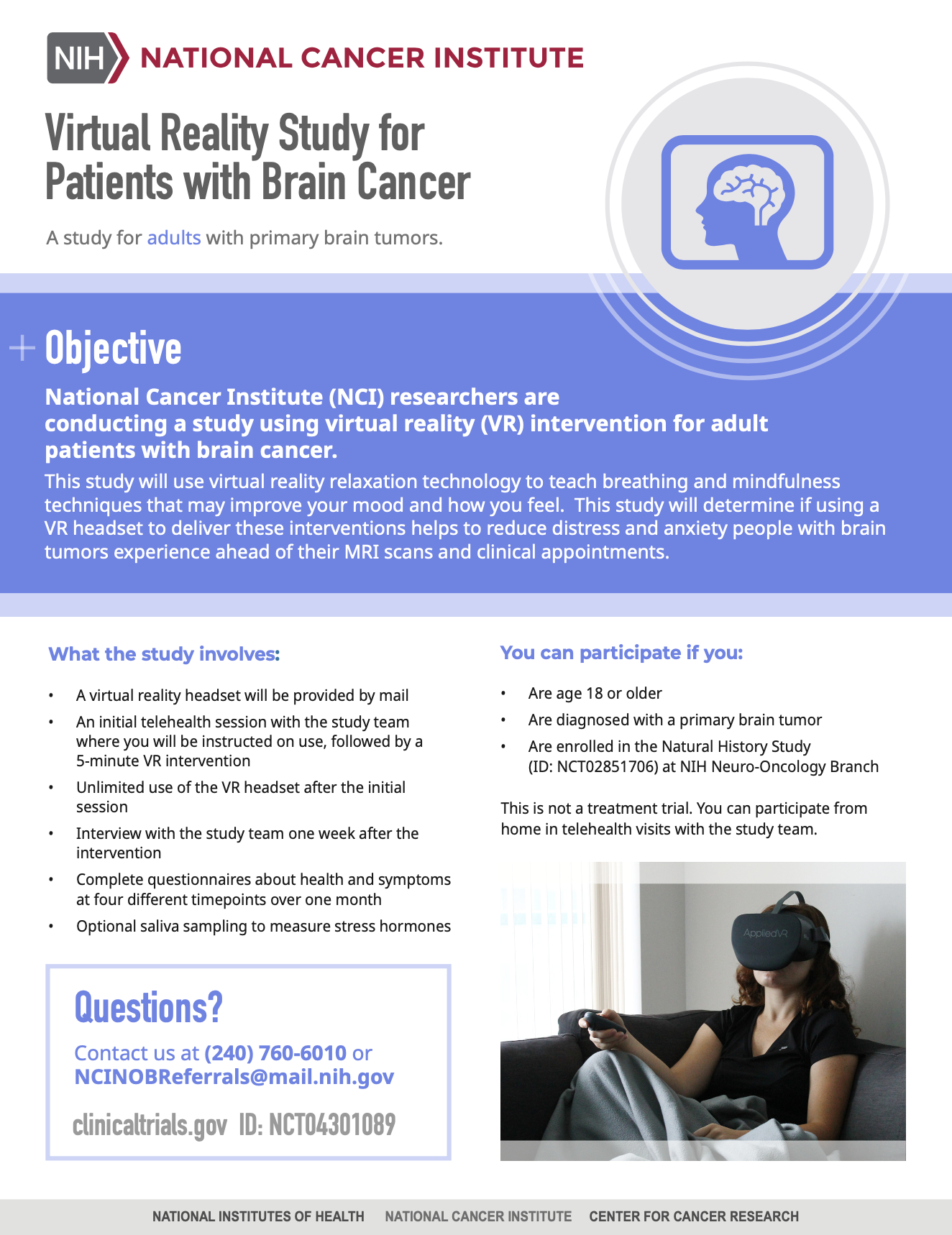


**Supplemental Figure 1.** **Recruitment flyer for VR study.** Flyer highlighting key aspects of VR study for distribution to potential patients recruited from clinic, via email, or through social media platforms.


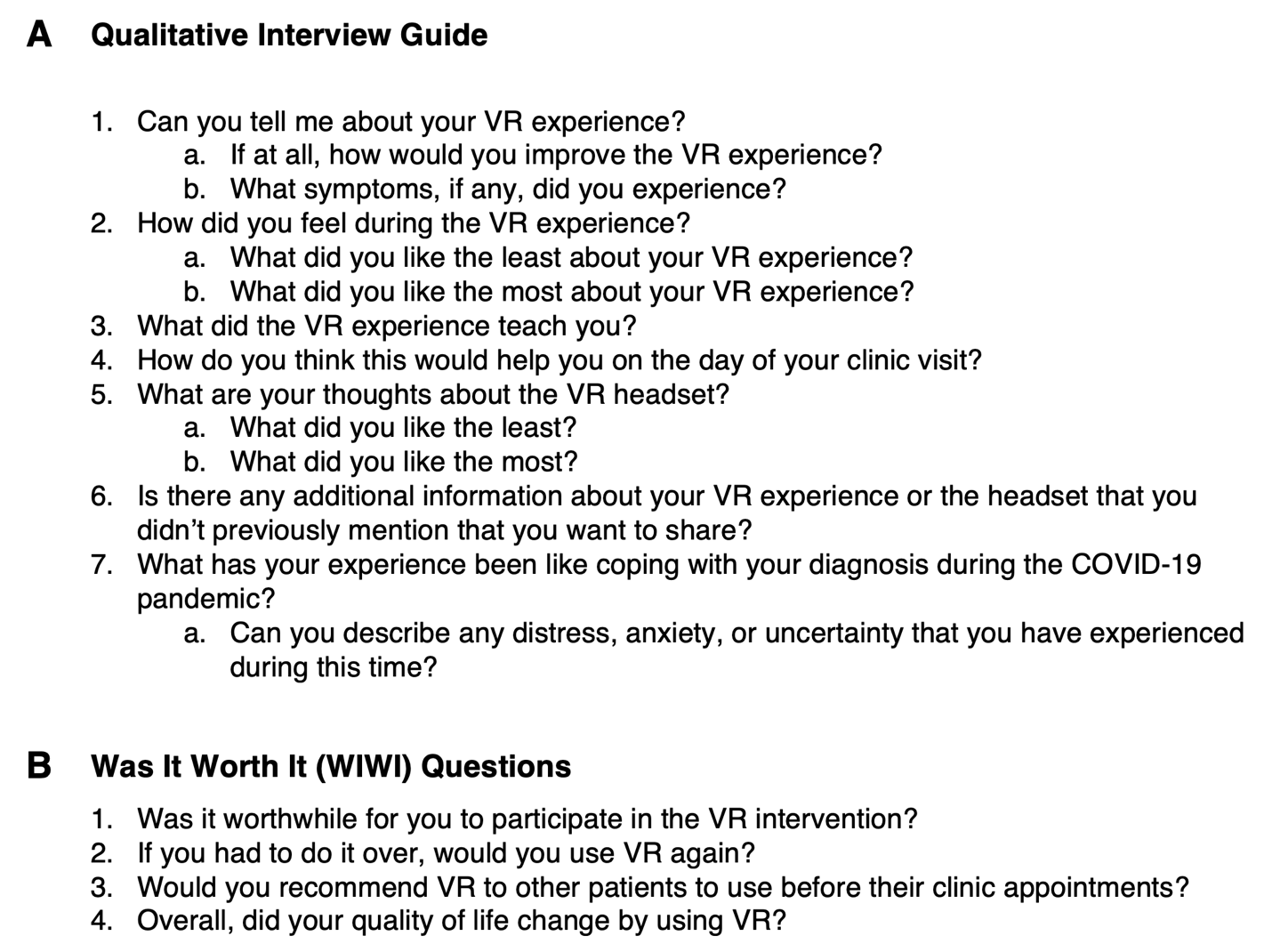


**Supplementary Figure 2. Qualitative assessment of patient satisfaction with VR intervention.** Semi-structured interview guide utilized during qualitative phone interview assessment for the VR study, including patient satisfaction with the intervention, lessons learned and feedback, and coping with their diagnosis during the COVID-19 pandemic.
